# (2-Carboxy­benzoato-κ^2^ *O* ^1^,*O* ^1′^)(5,5,7,12,12,14-hexa­methyl-1,4,8,11-tetra­azacyclo­tetra­decane-κ^4^ *N*)nickel(II) perchlorate monohydrate

**DOI:** 10.1107/S1600536809020169

**Published:** 2009-06-06

**Authors:** Guang-Chuan Ou, Min Zhang, Xian-You Yuan

**Affiliations:** a Department of Biology and Chemistry, Hunan University of Science and Engineering, Yongzhou Hunan 425100, People’s Republic of China

## Abstract

The title compound, [Ni(C_8_H_5_O_4_)(C_16_H_36_N_4_)]ClO_4_·H_2_O, has the Ni^II^ atom in a distorted octa­hedral geometry, surrounded by coordination by four N atoms of the 5,5,7,12,12,14-hexa­methyl-1,4,8,11-tetra­azacyclo­tetra­decane ligand in a folded configuration, and two carboxyl­ate O atoms of the 2-carboxy­benzoate ligand in *cis* positions. The complex cation, the disordered perchlorate anion [occupancies 0.639 (8):0.361 (8)] and uncoordinated water mol­ecules engage in N—H⋯O and O—H⋯O hydrogen bonding, forming a layer structure parallel to (010).

## Related literature

For background literature, see: Tait & Busch (1976 ▶); Curtis (1965 ▶). For related crystal structures, see: Zeigerson *et al.* (1982 ▶); Gao *et al.* (2002 ▶); Burrows *et al.* (2004 ▶); Ou *et al.* (2008 ▶). For a discussion of helical coordination polymers, see: Khatua *et al.* (2006 ▶); Lonnon *et al.* (2006 ▶); Telfer & Kuroda (2005 ▶).
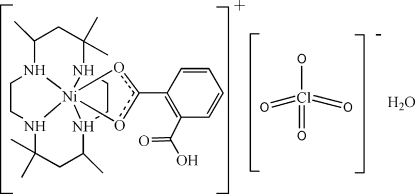


## Experimental

### 

#### Crystal data

[Ni(C_8_H_5_O_4_)(C_16_H_36_N_4_)]ClO_4_·H_2_O
*M* *_r_* = 625.78
Monoclinic, 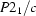

*a* = 9.7941 (12) Å
*b* = 17.354 (2) Å
*c* = 17.619 (2) Å
β = 102.105 (2)°
*V* = 2928.2 (6) Å^3^
*Z* = 4
Mo *K*α radiation
μ = 0.81 mm^−1^
*T* = 173 K
0.46 × 0.41 × 0.18 mm

#### Data collection

Bruker SMART CCD area-detector diffractometer
Absorption correction: multi-scan (*SADABS*; Sheldrick, 1996 ▶) *T*_min_ = 0.707, *T*_max_ = 0.868
14639 measured reflections
6322 independent reflections
4597 reflections with *I* > 2σ(*I*)
*R*_int_ = 0.030

#### Refinement

*R*[*F* ^2^ > 2σ(*F* ^2^)] = 0.048
*wR*(*F* ^2^) = 0.151
*S* = 1.07
6322 reflections
411 parameters
47 restraints
H atoms treated by a mixture of independent and constrained refinement
Δρ_max_ = 0.77 e Å^−3^
Δρ_min_ = −0.42 e Å^−3^

### 

Data collection: *SMART* (Bruker, 1999 ▶); cell refinement: *SAINT-Plus* (Bruker, 1999 ▶); data reduction: *SAINT-Plus*; program(s) used to solve structure: *SHELXS97* (Sheldrick, 2008 ▶); program(s) used to refine structure: *SHELXL97* (Sheldrick, 2008 ▶); molecular graphics: *SHELXTL* (Sheldrick, 2008 ▶); software used to prepare material for publication: *SHELXTL*.

## Supplementary Material

Crystal structure: contains datablocks I, global. DOI: 10.1107/S1600536809020169/ng2586sup1.cif

Structure factors: contains datablocks I. DOI: 10.1107/S1600536809020169/ng2586Isup2.hkl

Additional supplementary materials:  crystallographic information; 3D view; checkCIF report

## Figures and Tables

**Table 1:** Hydrogen-bond geometry (Å, °)

*D*—H⋯*A*	*D*—H	H⋯*A*	*D*⋯*A*	*D*—H⋯*A*
N1—H1*C*⋯O5^i^	0.93	2.29	3.094 (9)	144
N2—H2*C*⋯O6′^ii^	0.93	2.03	2.952 (10)	173
O4—H4⋯O1*W*	0.84	1.76	2.572 (4)	162
O1*W*—H2*W*⋯O8	0.855 (11)	2.13 (3)	2.827 (5)	138 (4)
O1*W*—H1*W*⋯O1^iii^	0.860 (11)	1.892 (16)	2.734 (3)	166 (4)

Symmetry codes: (i) 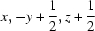; (ii) 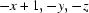; (iii) 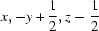.

**Fig. 1.**
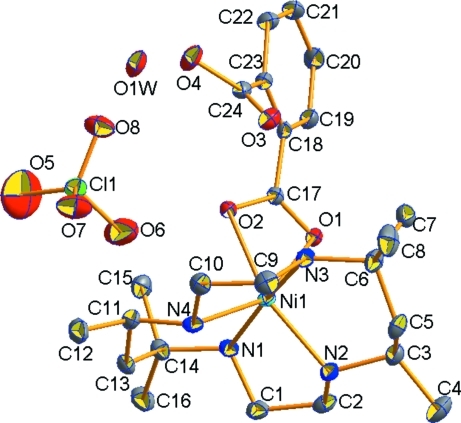
The molecular structure of (I), showing displacement ellipsoids at the 30% probability level. H-atoms have been excluded for clarity.

**Fig. 2.**
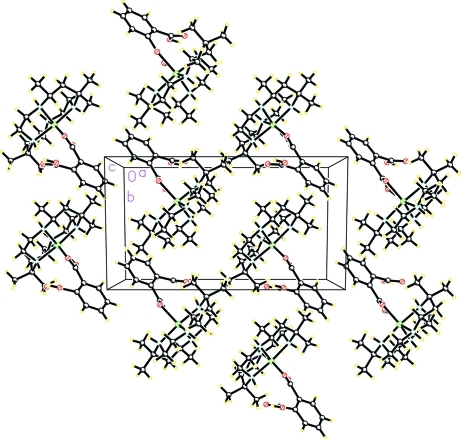
A view of the packing of the title compound.

## supplementary crystallographic information

### Comment

Recently, many helical structures were constructed through the coordination interactions, and helical polymers constructed *via* hydrogen bonding are still rare, and only a few cases have been reported (Khatua *et al.*, 2006; Lonnon *et al.*, 2006; Telfer & Kuroda, 2005). Then we employ racemic nickel(II) complex and phthalic acid as building blocks to construct helical structure, but the result of experiment indicate a racemic complex of [Ni(rac-*L*)(Hpt)(ClO_4_)]^.^H_2_O (pt=phthalic acid) is obtained.

In the asymmetric unit of (I), contains one [Ni(rac-*L*)(Hpt)]^+^ cation, one [ClO_4_]^-^ anion and one water molecule. The [ClO_4_]^-^ anion is disordered over two symmetry related sites with 50% occupancy. As illustrated in Fig.1, The six-coordinated Ni^2+^ of [Ni(rac-*L*)(Hpt)]^+^ cation displays a distorted octahedral geometry by coordination with four N atoms of macrocyclic ligand *L* in a folded configuration, and two carboxylate oxygen atoms of phthalic acid in *cis*-position. Neighbouring cations, anions and water molecule are discrete, connected to each other through intermolecular hydrogen bond.

### Experimental

Phthalic acid (H_2_pt, 0.166 g, 1 mmol) was mixed with NaOH (0.040 g, 1 mmol) dissolved in 10 ml of water. To this solution was added [Ni(rac-*L*)](ClO_4_)_2_ (0.541 g, 1 mmol) dissolved in a minimum amount of CH_3_CN. The solution was left to stand at room temperature and blue crystals formed after several weeks.

### Refinement

H atoms bound to C, O and N atoms were positioned geometrically and refined using the riding model, and with C—H = 0.95 to 1.00 Å, O—H = 0.84 Å and N—H = 0.93 Å, and with *U*(H) set to 1.2 to 1.5 *U*_eq_(C, O, N).

H atoms attached to O (water) atoms were located in difference Fourier maps and condtrained to ride on their carrier atoms, with O—H distances in the range 0.86 Å, and with *U*_iso_ (H) = 1.2 times *U*_eq_ (O).

Disorder in the [ClO_4_]^-^ anion required the Cl–O distance to be restrained to 1.44±0.01 Å and the O–O distance to 2.35±0.02 Å.

### Crystal data

**Table:**

[Ni(C_8_H_5_O_4_)(C_16_H_36_N_4_)]ClO_4_·H_2_O	*F*(000) = 1328
*M**_r_* = 625.78	*D*_x_ = 1.420 Mg m^−^^3^
Monoclinic, *P*2_1_/*c*	Mo *K*α radiation, λ = 0.71073 Å
*a* = 9.7941 (12) Å	Cell parameters from 5283 reflections
*b* = 17.354 (2) Å	θ = 2.4–27.0°
*c* = 17.619 (2) Å	µ = 0.81 mm^−^^1^
β = 102.105 (2)°	*T* = 173 K
*V* = 2928.2 (6) Å^3^	Block, blue
*Z* = 4	0.46 × 0.41 × 0.18 mm

### Data collection

**Table:**

Bruker SMART CCD area-detector diffractometer	6322 independent reflections
Radiation source: fine-focus sealed tube	4597 reflections with *I* > 2σ(*I*)
graphite	*R*_int_ = 0.030
φ and ω scans	θ_max_ = 27.1°, θ_min_ = 1.7°
Absorption correction: multi-scan (*SADABS*; Sheldrick, 1996)	*h* = −12→11
*T*_min_ = 0.707, *T*_max_ = 0.868	*k* = −22→17
14639 measured reflections	*l* = −22→21

### Refinement

**Table:**

Refinement on *F*^2^	Primary atom site location: structure-invariant direct methods
Least-squares matrix: full	Secondary atom site location: difference Fourier map
*R*[*F*^2^ > 2σ(*F*^2^)] = 0.048	Hydrogen site location: inferred from neighbouring sites
*wR*(*F*^2^) = 0.151	H atoms treated by a mixture of independent and constrained refinement
*S* = 1.07	*w* = 1/[σ^2^(*F*_o_^2^) + (0.0858*P*)^2^ + 1.13*P*] where *P* = (*F*_o_^2^ + 2*F*_c_^2^)/3
6322 reflections	(Δ/σ)_max_ < 0.001
411 parameters	Δρ_max_ = 0.77 e Å^−^^3^
47 restraints	Δρ_min_ = −0.42 e Å^−^^3^

### Special details

**Table:**

Geometry. All e.s.d.'s (except the e.s.d. in the dihedral angle between two l.s. planes) are estimated using the full covariance matrix. The cell e.s.d.'s are taken into account individually in the estimation of e.s.d.'s in distances, angles and torsion angles; correlations between e.s.d.'s in cell parameters are only used when they are defined by crystal symmetry. An approximate (isotropic) treatment of cell e.s.d.'s is used for estimating e.s.d.'s involving l.s. planes.
Refinement. Refinement of *F*^2^ against ALL reflections. The weighted *R*-factor *wR* and goodness of fit *S* are based on *F*^2^, conventional *R*-factors *R* are based on *F*, with *F* set to zero for negative *F*^2^. The threshold expression of *F*^2^ > σ(*F*^2^) is used only for calculating *R*-factors(gt) *etc*. and is not relevant to the choice of reflections for refinement. *R*-factors based on *F*^2^ are statistically about twice as large as those based on *F*, and *R*- factors based on ALL data will be even larger.

### Fractional atomic coordinates and isotropic or equivalent isotropic displacement parameters (Å^2^)

**Table:**

	*x*	*y*	*z*	*U*_iso_*/*U*_eq_	Occ. (<1)
Ni1	0.32081 (4)	0.21532 (2)	0.13185 (2)	0.02393 (14)	
O1	0.1559 (2)	0.29722 (12)	0.13918 (12)	0.0279 (5)	
O2	0.2165 (2)	0.27282 (12)	0.02880 (13)	0.0277 (5)	
N1	0.4733 (3)	0.30047 (15)	0.17667 (16)	0.0292 (6)	
H1C	0.4229	0.3432	0.1871	0.035*	
O3	−0.0163 (3)	0.18524 (14)	−0.05475 (14)	0.0379 (6)	
N3	0.1821 (3)	0.12179 (15)	0.09356 (16)	0.0307 (6)	
H3A	0.1206	0.1395	0.0495	0.037*	
N4	0.4586 (3)	0.15922 (15)	0.07460 (16)	0.0291 (6)	
H4D	0.5215	0.1309	0.1109	0.035*	
C17	0.1329 (3)	0.30254 (17)	0.06606 (19)	0.0257 (7)	
C18	0.0067 (3)	0.34386 (17)	0.02248 (18)	0.0251 (6)	
N2	0.3653 (3)	0.17186 (16)	0.24485 (16)	0.0313 (6)	
H2C	0.4281	0.1314	0.2465	0.038*	
C23	−0.0603 (3)	0.32076 (19)	−0.05238 (19)	0.0282 (7)	
O4	−0.0144 (3)	0.25603 (16)	−0.15998 (15)	0.0474 (7)	
H4	0.0042	0.2136	−0.1784	0.071*	
C11	0.5392 (4)	0.2112 (2)	0.0331 (2)	0.0342 (8)	
H11	0.4714	0.2448	−0.0025	0.041*	
C24	−0.0246 (3)	0.2465 (2)	−0.08727 (19)	0.0306 (7)	
C3	0.2428 (4)	0.1426 (2)	0.2747 (2)	0.0381 (8)	
H3	0.1712	0.1845	0.2687	0.046*	
C1	0.5447 (4)	0.2708 (2)	0.2534 (2)	0.0387 (8)	
H1A	0.5936	0.3134	0.2854	0.046*	
H1B	0.6150	0.2317	0.2468	0.046*	
C2	0.4381 (4)	0.2353 (2)	0.2933 (2)	0.0395 (9)	
H2A	0.4850	0.2150	0.3447	0.047*	
H2B	0.3697	0.2748	0.3013	0.047*	
C22	−0.1695 (4)	0.3647 (2)	−0.0938 (2)	0.0392 (8)	
H22	−0.2133	0.3500	−0.1451	0.047*	
C9	0.2719 (4)	0.0646 (2)	0.0655 (2)	0.0399 (8)	
H9A	0.2131	0.0271	0.0308	0.048*	
H9B	0.3274	0.0360	0.1101	0.048*	
C13	0.6348 (4)	0.2627 (2)	0.0907 (2)	0.0358 (8)	
H13A	0.7054	0.2845	0.0639	0.043*	
H13B	0.6854	0.2294	0.1330	0.043*	
C19	−0.0391 (4)	0.40969 (19)	0.0544 (2)	0.0337 (8)	
H19	0.0059	0.4259	0.1050	0.040*	
C14	0.5715 (4)	0.3301 (2)	0.1285 (2)	0.0355 (8)	
C4	0.2814 (6)	0.1203 (3)	0.3605 (2)	0.0602 (12)	
H4A	0.3532	0.0800	0.3679	0.090*	
H4B	0.1983	0.1009	0.3771	0.090*	
H4C	0.3174	0.1655	0.3916	0.090*	
C7	−0.0259 (4)	0.1456 (2)	0.1469 (2)	0.0433 (9)	
H7A	0.0142	0.1958	0.1648	0.065*	
H7B	−0.0835	0.1270	0.1823	0.065*	
H7C	−0.0837	0.1509	0.0946	0.065*	
C20	−0.1508 (4)	0.4520 (2)	0.0127 (2)	0.0415 (9)	
H20	−0.1829	0.4964	0.0353	0.050*	
C10	0.3674 (4)	0.1046 (2)	0.0226 (2)	0.0363 (8)	
H10A	0.4255	0.0662	0.0024	0.044*	
H10B	0.3117	0.1329	−0.0222	0.044*	
C15	0.4879 (4)	0.3840 (2)	0.0673 (2)	0.0425 (9)	
H15A	0.4044	0.3572	0.0392	0.064*	
H15B	0.5457	0.3994	0.0307	0.064*	
H15C	0.4601	0.4299	0.0928	0.064*	
C21	−0.2147 (4)	0.4296 (2)	−0.0612 (2)	0.0446 (9)	
H21	−0.2901	0.4590	−0.0898	0.053*	
C5	0.1784 (4)	0.0737 (2)	0.2264 (2)	0.0419 (9)	
H5A	0.2552	0.0383	0.2213	0.050*	
H5B	0.1186	0.0460	0.2564	0.050*	
C6	0.0915 (4)	0.0883 (2)	0.1453 (2)	0.0378 (8)	
C16	0.6912 (4)	0.3766 (3)	0.1778 (3)	0.0499 (10)	
H16A	0.6527	0.4155	0.2078	0.075*	
H16B	0.7446	0.4020	0.1438	0.075*	
H16C	0.7527	0.3419	0.2134	0.075*	
C12	0.6241 (4)	0.1662 (3)	−0.0161 (2)	0.0482 (10)	
H12A	0.6838	0.1286	0.0168	0.072*	
H12B	0.6823	0.2020	−0.0384	0.072*	
H12C	0.5603	0.1392	−0.0579	0.072*	
C8	0.0254 (5)	0.0123 (2)	0.1121 (3)	0.0574 (12)	
H8A	−0.0166	0.0190	0.0569	0.086*	
H8B	−0.0470	−0.0030	0.1400	0.086*	
H8C	0.0973	−0.0278	0.1181	0.086*	
O1W	0.0544 (3)	0.14387 (16)	−0.23970 (16)	0.0481 (7)	
H2W	0.118 (3)	0.111 (2)	−0.221 (2)	0.058*	
H1W	0.072 (4)	0.161 (2)	−0.2823 (14)	0.058*	
Cl1	0.3438 (6)	−0.0040 (3)	−0.2176 (3)	0.0381 (10)	0.639 (8)
Cl1'	0.3673 (10)	0.0103 (6)	−0.2158 (6)	0.0362 (18)	0.361 (8)
O5	0.4344 (13)	0.0455 (7)	−0.2420 (9)	0.203 (6)	0.639 (8)
O6	0.3623 (8)	−0.0075 (5)	−0.1357 (3)	0.110 (3)	0.639 (8)
O7	0.3697 (8)	−0.0818 (3)	−0.2264 (4)	0.073 (2)	0.639 (8)
O8	0.2001 (5)	0.0061 (3)	−0.2550 (3)	0.0588 (18)	0.639 (8)
O5'	0.2976 (14)	0.0479 (5)	−0.2866 (5)	0.088 (5)	0.361 (8)
O6'	0.4169 (15)	−0.0535 (8)	−0.2485 (9)	0.101 (5)	0.361 (8)
O7'	0.4898 (15)	0.0527 (8)	−0.1817 (8)	0.152 (8)	0.361 (8)
O8'	0.2850 (14)	0.0248 (7)	−0.1601 (7)	0.098 (4)	0.361 (8)

### Atomic displacement parameters (Å^2^)

**Table:**

	*U*^11^	*U*^22^	*U*^33^	*U*^12^	*U*^13^	*U*^23^
Ni1	0.0271 (2)	0.0244 (2)	0.0195 (2)	0.00258 (16)	0.00305 (15)	−0.00112 (15)
O1	0.0295 (12)	0.0330 (12)	0.0216 (11)	0.0013 (9)	0.0061 (9)	0.0000 (9)
O2	0.0262 (12)	0.0310 (11)	0.0264 (12)	0.0036 (9)	0.0067 (9)	0.0019 (9)
N1	0.0267 (14)	0.0304 (14)	0.0297 (15)	0.0014 (11)	0.0040 (11)	−0.0041 (11)
O3	0.0481 (15)	0.0336 (12)	0.0308 (13)	−0.0032 (11)	0.0052 (11)	0.0010 (11)
N3	0.0349 (16)	0.0295 (14)	0.0276 (14)	−0.0039 (12)	0.0066 (12)	0.0000 (11)
N4	0.0297 (15)	0.0295 (13)	0.0263 (14)	0.0020 (11)	0.0013 (11)	−0.0028 (11)
C17	0.0266 (16)	0.0248 (15)	0.0257 (16)	−0.0030 (13)	0.0056 (13)	−0.0008 (12)
C18	0.0275 (16)	0.0261 (15)	0.0240 (15)	0.0011 (13)	0.0107 (13)	0.0055 (12)
N2	0.0353 (16)	0.0343 (15)	0.0246 (14)	0.0051 (12)	0.0072 (12)	0.0018 (12)
C23	0.0266 (17)	0.0320 (16)	0.0270 (16)	0.0019 (13)	0.0075 (13)	0.0052 (13)
O4	0.070 (2)	0.0463 (15)	0.0302 (14)	0.0120 (14)	0.0210 (13)	0.0026 (12)
C11	0.0335 (19)	0.0417 (19)	0.0280 (17)	0.0011 (15)	0.0082 (14)	−0.0003 (15)
C24	0.0273 (17)	0.0380 (18)	0.0255 (17)	0.0003 (14)	0.0031 (13)	0.0011 (14)
C3	0.046 (2)	0.0404 (19)	0.0307 (18)	0.0032 (16)	0.0146 (16)	0.0054 (15)
C1	0.038 (2)	0.046 (2)	0.0284 (18)	0.0028 (17)	0.0008 (15)	−0.0055 (15)
C2	0.048 (2)	0.044 (2)	0.0223 (17)	0.0004 (17)	−0.0005 (15)	−0.0046 (15)
C22	0.037 (2)	0.047 (2)	0.0315 (19)	0.0065 (17)	0.0015 (15)	0.0058 (16)
C9	0.047 (2)	0.0314 (17)	0.041 (2)	−0.0025 (16)	0.0076 (17)	−0.0084 (15)
C13	0.0241 (17)	0.0445 (19)	0.040 (2)	−0.0014 (15)	0.0104 (15)	−0.0005 (16)
C19	0.040 (2)	0.0340 (17)	0.0293 (18)	0.0055 (15)	0.0119 (15)	0.0038 (14)
C14	0.0290 (18)	0.0378 (19)	0.040 (2)	−0.0038 (15)	0.0074 (15)	−0.0027 (15)
C4	0.083 (3)	0.066 (3)	0.032 (2)	−0.004 (2)	0.011 (2)	0.011 (2)
C7	0.038 (2)	0.051 (2)	0.043 (2)	−0.0073 (17)	0.0136 (17)	0.0032 (18)
C20	0.045 (2)	0.041 (2)	0.043 (2)	0.0164 (17)	0.0174 (17)	0.0065 (17)
C10	0.039 (2)	0.0370 (18)	0.0336 (19)	0.0012 (15)	0.0087 (15)	−0.0113 (15)
C15	0.045 (2)	0.0372 (19)	0.046 (2)	−0.0041 (17)	0.0120 (18)	0.0066 (17)
C21	0.038 (2)	0.052 (2)	0.043 (2)	0.0179 (18)	0.0095 (17)	0.0129 (18)
C5	0.058 (3)	0.0343 (18)	0.036 (2)	−0.0039 (17)	0.0169 (18)	0.0123 (16)
C6	0.046 (2)	0.0337 (18)	0.0355 (19)	−0.0096 (16)	0.0123 (16)	0.0036 (15)
C16	0.037 (2)	0.057 (2)	0.055 (3)	−0.0129 (19)	0.0075 (18)	−0.009 (2)
C12	0.043 (2)	0.066 (3)	0.039 (2)	−0.003 (2)	0.0167 (18)	−0.0105 (19)
C8	0.067 (3)	0.049 (2)	0.062 (3)	−0.026 (2)	0.027 (2)	−0.007 (2)
O1W	0.069 (2)	0.0448 (16)	0.0372 (15)	0.0128 (14)	0.0273 (14)	0.0081 (12)
Cl1	0.0404 (19)	0.0348 (18)	0.0393 (12)	0.0025 (14)	0.0087 (11)	0.0016 (10)
Cl1'	0.035 (3)	0.034 (3)	0.040 (2)	0.005 (3)	0.0083 (19)	0.006 (2)
O5	0.180 (9)	0.197 (9)	0.234 (10)	−0.089 (7)	0.044 (8)	0.110 (8)
O6	0.092 (5)	0.180 (7)	0.054 (4)	0.050 (5)	0.010 (3)	−0.003 (4)
O7	0.068 (5)	0.056 (4)	0.086 (5)	0.017 (3)	−0.006 (3)	0.009 (3)
O8	0.045 (3)	0.063 (3)	0.063 (3)	0.016 (2)	0.000 (2)	−0.012 (3)
O5'	0.124 (11)	0.046 (5)	0.069 (7)	0.021 (6)	−0.040 (7)	0.004 (5)
O6'	0.085 (8)	0.090 (8)	0.109 (9)	0.056 (7)	−0.025 (6)	−0.050 (7)
O7'	0.27 (2)	0.110 (11)	0.073 (9)	−0.111 (13)	0.018 (11)	−0.030 (8)
O8'	0.121 (9)	0.099 (7)	0.080 (7)	0.041 (6)	0.033 (7)	−0.005 (6)

### Geometric parameters (Å, °)

**Table:**

Ni1—N4	2.087 (3)	C13—C14	1.539 (5)
Ni1—N2	2.088 (3)	C13—H13A	0.9900
Ni1—N1	2.131 (3)	C13—H13B	0.9900
Ni1—N3	2.133 (3)	C19—C20	1.393 (5)
Ni1—O2	2.135 (2)	C19—H19	0.9500
Ni1—O1	2.176 (2)	C14—C15	1.529 (5)
Ni1—C17	2.475 (3)	C14—C16	1.533 (5)
O1—C17	1.264 (4)	C4—H4A	0.9800
O2—C17	1.262 (4)	C4—H4B	0.9800
N1—C1	1.478 (4)	C4—H4C	0.9800
N1—C14	1.501 (4)	C7—C6	1.524 (5)
N1—H1C	0.9300	C7—H7A	0.9800
O3—C24	1.202 (4)	C7—H7B	0.9800
N3—C9	1.479 (4)	C7—H7C	0.9800
N3—C6	1.516 (4)	C20—C21	1.378 (6)
N3—H3A	0.9300	C20—H20	0.9500
N4—C10	1.480 (4)	C10—H10A	0.9900
N4—C11	1.488 (4)	C10—H10B	0.9900
N4—H4D	0.9300	C15—H15A	0.9800
C17—C18	1.495 (4)	C15—H15B	0.9800
C18—C19	1.389 (4)	C15—H15C	0.9800
C18—C23	1.403 (4)	C21—H21	0.9500
N2—C2	1.481 (4)	C5—C6	1.523 (5)
N2—C3	1.496 (4)	C5—H5A	0.9900
N2—H2C	0.9300	C5—H5B	0.9900
C23—C22	1.389 (5)	C6—C8	1.531 (5)
C23—C24	1.501 (5)	C16—H16A	0.9800
O4—C24	1.316 (4)	C16—H16B	0.9800
O4—H4	0.8400	C16—H16C	0.9800
C11—C13	1.520 (5)	C12—H12A	0.9800
C11—C12	1.534 (5)	C12—H12B	0.9800
C11—H11	1.0000	C12—H12C	0.9800
C3—C5	1.527 (5)	C8—H8A	0.9800
C3—C4	1.530 (5)	C8—H8B	0.9800
C3—H3	1.0000	C8—H8C	0.9800
C1—C2	1.508 (5)	O1W—H2W	0.855 (11)
C1—H1A	0.9900	O1W—H1W	0.860 (11)
C1—H1B	0.9900	Cl1—O5	1.367 (7)
C2—H2A	0.9900	Cl1—O7	1.390 (7)
C2—H2B	0.9900	Cl1—O6	1.418 (7)
C22—C21	1.379 (5)	Cl1—O8	1.434 (6)
C22—H22	0.9500	Cl1'—O6'	1.383 (9)
C9—C10	1.492 (5)	Cl1'—O8'	1.416 (9)
C9—H9A	0.9900	Cl1'—O7'	1.428 (9)
C9—H9B	0.9900	Cl1'—O5'	1.446 (9)
			
N4—Ni1—N2	105.40 (11)	C10—C9—H9A	109.7
N4—Ni1—N1	91.66 (10)	N3—C9—H9B	109.7
N2—Ni1—N1	84.67 (11)	C10—C9—H9B	109.7
N4—Ni1—N3	85.73 (11)	H9A—C9—H9B	108.2
N2—Ni1—N3	91.06 (11)	C11—C13—C14	119.2 (3)
N1—Ni1—N3	174.23 (11)	C11—C13—H13A	107.5
N4—Ni1—O2	92.84 (10)	C14—C13—H13A	107.5
N2—Ni1—O2	161.23 (10)	C11—C13—H13B	107.5
N1—Ni1—O2	99.45 (10)	C14—C13—H13B	107.5
N3—Ni1—O2	85.83 (10)	H13A—C13—H13B	107.0
N4—Ni1—O1	153.72 (10)	C18—C19—C20	120.3 (3)
N2—Ni1—O1	100.86 (10)	C18—C19—H19	119.8
N1—Ni1—O1	89.79 (10)	C20—C19—H19	119.8
N3—Ni1—O1	94.84 (10)	N1—C14—C15	107.2 (3)
O2—Ni1—O1	61.07 (8)	N1—C14—C16	111.4 (3)
N4—Ni1—C17	123.46 (11)	C15—C14—C16	108.1 (3)
N2—Ni1—C17	130.81 (11)	N1—C14—C13	110.4 (3)
N1—Ni1—C17	98.26 (10)	C15—C14—C13	111.4 (3)
N3—Ni1—C17	87.46 (10)	C16—C14—C13	108.4 (3)
O2—Ni1—C17	30.65 (9)	C3—C4—H4A	109.5
O1—Ni1—C17	30.68 (9)	C3—C4—H4B	109.5
C17—O1—Ni1	87.86 (18)	H4A—C4—H4B	109.5
C17—O2—Ni1	89.74 (19)	C3—C4—H4C	109.5
C1—N1—C14	113.5 (3)	H4A—C4—H4C	109.5
C1—N1—Ni1	104.9 (2)	H4B—C4—H4C	109.5
C14—N1—Ni1	120.7 (2)	C6—C7—H7A	109.5
C1—N1—H1C	105.5	C6—C7—H7B	109.5
C14—N1—H1C	105.5	H7A—C7—H7B	109.5
Ni1—N1—H1C	105.5	C6—C7—H7C	109.5
C9—N3—C6	114.1 (3)	H7A—C7—H7C	109.5
C9—N3—Ni1	103.5 (2)	H7B—C7—H7C	109.5
C6—N3—Ni1	121.1 (2)	C21—C20—C19	120.1 (3)
C9—N3—H3A	105.7	C21—C20—H20	120.0
C6—N3—H3A	105.7	C19—C20—H20	120.0
Ni1—N3—H3A	105.7	N4—C10—C9	110.2 (3)
C10—N4—C11	113.4 (3)	N4—C10—H10A	109.6
C10—N4—Ni1	103.3 (2)	C9—C10—H10A	109.6
C11—N4—Ni1	114.7 (2)	N4—C10—H10B	109.6
C10—N4—H4D	108.4	C9—C10—H10B	109.6
C11—N4—H4D	108.4	H10A—C10—H10B	108.1
Ni1—N4—H4D	108.4	C14—C15—H15A	109.5
O2—C17—O1	120.3 (3)	C14—C15—H15B	109.5
O2—C17—C18	119.1 (3)	H15A—C15—H15B	109.5
O1—C17—C18	120.6 (3)	C14—C15—H15C	109.5
O2—C17—Ni1	59.61 (16)	H15A—C15—H15C	109.5
O1—C17—Ni1	61.46 (16)	H15B—C15—H15C	109.5
C18—C17—Ni1	171.0 (2)	C20—C21—C22	120.2 (3)
C19—C18—C23	119.3 (3)	C20—C21—H21	119.9
C19—C18—C17	119.3 (3)	C22—C21—H21	119.9
C23—C18—C17	121.3 (3)	C6—C5—C3	118.4 (3)
C2—N2—C3	112.0 (3)	C6—C5—H5A	107.7
C2—N2—Ni1	105.1 (2)	C3—C5—H5A	107.7
C3—N2—Ni1	115.8 (2)	C6—C5—H5B	107.7
C2—N2—H2C	107.9	C3—C5—H5B	107.7
C3—N2—H2C	107.9	H5A—C5—H5B	107.1
Ni1—N2—H2C	107.9	N3—C6—C5	110.2 (3)
C22—C23—C18	119.6 (3)	N3—C6—C7	107.5 (3)
C22—C23—C24	118.4 (3)	C5—C6—C7	111.5 (3)
C18—C23—C24	121.8 (3)	N3—C6—C8	110.7 (3)
C24—O4—H4	109.5	C5—C6—C8	108.9 (3)
N4—C11—C13	110.2 (3)	C7—C6—C8	108.0 (3)
N4—C11—C12	112.0 (3)	C14—C16—H16A	109.5
C13—C11—C12	110.2 (3)	C14—C16—H16B	109.5
N4—C11—H11	108.1	H16A—C16—H16B	109.5
C13—C11—H11	108.1	C14—C16—H16C	109.5
C12—C11—H11	108.1	H16A—C16—H16C	109.5
O3—C24—O4	124.3 (3)	H16B—C16—H16C	109.5
O3—C24—C23	124.2 (3)	C11—C12—H12A	109.5
O4—C24—C23	111.3 (3)	C11—C12—H12B	109.5
N2—C3—C5	109.8 (3)	H12A—C12—H12B	109.5
N2—C3—C4	112.5 (3)	C11—C12—H12C	109.5
C5—C3—C4	109.8 (3)	H12A—C12—H12C	109.5
N2—C3—H3	108.2	H12B—C12—H12C	109.5
C5—C3—H3	108.2	C6—C8—H8A	109.5
C4—C3—H3	108.2	C6—C8—H8B	109.5
N1—C1—C2	109.1 (3)	H8A—C8—H8B	109.5
N1—C1—H1A	109.9	C6—C8—H8C	109.5
C2—C1—H1A	109.9	H8A—C8—H8C	109.5
N1—C1—H1B	109.9	H8B—C8—H8C	109.5
C2—C1—H1B	109.9	H2W—O1W—H1W	108.1 (17)
H1A—C1—H1B	108.3	O5—Cl1—O7	115.5 (8)
N2—C2—C1	109.4 (3)	O5—Cl1—O6	113.2 (8)
N2—C2—H2A	109.8	O7—Cl1—O6	94.8 (6)
C1—C2—H2A	109.8	O5—Cl1—O8	114.8 (7)
N2—C2—H2B	109.8	O7—Cl1—O8	104.5 (5)
C1—C2—H2B	109.8	O6—Cl1—O8	112.1 (6)
H2A—C2—H2B	108.2	O6'—Cl1'—O8'	137.0 (11)
C21—C22—C23	120.5 (3)	O6'—Cl1'—O7'	104.5 (10)
C21—C22—H22	119.7	O8'—Cl1'—O7'	99.6 (9)
C23—C22—H22	119.7	O6'—Cl1'—O5'	98.4 (9)
N3—C9—C10	109.7 (3)	O8'—Cl1'—O5'	106.4 (9)
N3—C9—H9A	109.7	O7'—Cl1'—O5'	109.5 (9)
			
N4—Ni1—O1—C17	13.4 (3)	O1—C17—C18—C23	−147.6 (3)
N2—Ni1—O1—C17	−168.73 (18)	Ni1—C17—C18—C23	−46.7 (16)
N1—Ni1—O1—C17	106.74 (19)	N4—Ni1—N2—C2	107.1 (2)
N3—Ni1—O1—C17	−76.71 (19)	N1—Ni1—N2—C2	16.9 (2)
O2—Ni1—O1—C17	5.81 (17)	N3—Ni1—N2—C2	−167.0 (2)
N4—Ni1—O2—C17	177.56 (18)	O2—Ni1—N2—C2	−86.9 (4)
N2—Ni1—O2—C17	11.1 (4)	O1—Ni1—N2—C2	−71.9 (2)
N1—Ni1—O2—C17	−90.29 (19)	C17—Ni1—N2—C2	−79.5 (3)
N3—Ni1—O2—C17	92.06 (19)	N4—Ni1—N2—C3	−128.8 (2)
O1—Ni1—O2—C17	−5.81 (17)	N1—Ni1—N2—C3	141.0 (2)
N4—Ni1—N1—C1	−92.5 (2)	N3—Ni1—N2—C3	−42.9 (2)
N2—Ni1—N1—C1	12.8 (2)	O2—Ni1—N2—C3	37.2 (4)
N3—Ni1—N1—C1	−29.6 (12)	O1—Ni1—N2—C3	52.2 (2)
O2—Ni1—N1—C1	174.4 (2)	C17—Ni1—N2—C3	44.6 (3)
O1—Ni1—N1—C1	113.8 (2)	C19—C18—C23—C22	1.2 (5)
C17—Ni1—N1—C1	143.4 (2)	C17—C18—C23—C22	−174.2 (3)
N4—Ni1—N1—C14	37.3 (2)	C19—C18—C23—C24	−174.3 (3)
N2—Ni1—N1—C14	142.6 (2)	C17—C18—C23—C24	10.3 (5)
N3—Ni1—N1—C14	100.2 (11)	C10—N4—C11—C13	−177.4 (3)
O2—Ni1—N1—C14	−55.9 (2)	Ni1—N4—C11—C13	64.2 (3)
O1—Ni1—N1—C14	−116.5 (2)	C10—N4—C11—C12	−54.3 (4)
C17—Ni1—N1—C14	−86.9 (2)	Ni1—N4—C11—C12	−172.7 (2)
N4—Ni1—N3—C9	12.3 (2)	C22—C23—C24—O3	−126.8 (4)
N2—Ni1—N3—C9	−93.1 (2)	C18—C23—C24—O3	48.7 (5)
N1—Ni1—N3—C9	−50.9 (11)	C22—C23—C24—O4	48.2 (4)
O2—Ni1—N3—C9	105.5 (2)	C18—C23—C24—O4	−136.3 (3)
O1—Ni1—N3—C9	165.9 (2)	C2—N2—C3—C5	−175.8 (3)
C17—Ni1—N3—C9	136.1 (2)	Ni1—N2—C3—C5	63.8 (3)
N4—Ni1—N3—C6	141.7 (3)	C2—N2—C3—C4	−53.1 (4)
N2—Ni1—N3—C6	36.3 (3)	Ni1—N2—C3—C4	−173.6 (3)
N1—Ni1—N3—C6	78.5 (11)	C14—N1—C1—C2	−174.3 (3)
O2—Ni1—N3—C6	−125.2 (2)	Ni1—N1—C1—C2	−40.4 (3)
O1—Ni1—N3—C6	−64.7 (2)	C3—N2—C2—C1	−170.7 (3)
C17—Ni1—N3—C6	−94.5 (3)	Ni1—N2—C2—C1	−44.2 (3)
N2—Ni1—N4—C10	107.1 (2)	N1—C1—C2—N2	59.3 (4)
N1—Ni1—N4—C10	−167.9 (2)	C18—C23—C22—C21	−1.7 (5)
N3—Ni1—N4—C10	17.2 (2)	C24—C23—C22—C21	174.0 (3)
O2—Ni1—N4—C10	−68.4 (2)	C6—N3—C9—C10	−173.9 (3)
O1—Ni1—N4—C10	−75.1 (3)	Ni1—N3—C9—C10	−40.4 (3)
C17—Ni1—N4—C10	−66.9 (2)	N4—C11—C13—C14	−74.1 (4)
N2—Ni1—N4—C11	−128.9 (2)	C12—C11—C13—C14	161.7 (3)
N1—Ni1—N4—C11	−44.0 (2)	C23—C18—C19—C20	0.3 (5)
N3—Ni1—N4—C11	141.1 (2)	C17—C18—C19—C20	175.7 (3)
O2—Ni1—N4—C11	55.5 (2)	C1—N1—C14—C15	−159.6 (3)
O1—Ni1—N4—C11	48.9 (3)	Ni1—N1—C14—C15	74.6 (3)
C17—Ni1—N4—C11	57.0 (2)	C1—N1—C14—C16	−41.5 (4)
Ni1—O2—C17—O1	10.2 (3)	Ni1—N1—C14—C16	−167.3 (2)
Ni1—O2—C17—C18	−169.7 (2)	C1—N1—C14—C13	79.0 (3)
Ni1—O1—C17—O2	−10.0 (3)	Ni1—N1—C14—C13	−46.9 (3)
Ni1—O1—C17—C18	169.9 (3)	C11—C13—C14—N1	62.9 (4)
N4—Ni1—C17—O2	−2.9 (2)	C11—C13—C14—C15	−56.1 (4)
N2—Ni1—C17—O2	−175.31 (17)	C11—C13—C14—C16	−174.9 (3)
N1—Ni1—C17—O2	94.60 (18)	C18—C19—C20—C21	−1.3 (6)
N3—Ni1—C17—O2	−86.11 (18)	C11—N4—C10—C9	−169.9 (3)
O1—Ni1—C17—O2	170.0 (3)	Ni1—N4—C10—C9	−45.1 (3)
N4—Ni1—C17—O1	−172.92 (16)	N3—C9—C10—N4	61.0 (4)
N2—Ni1—C17—O1	14.7 (2)	C19—C20—C21—C22	0.7 (6)
N1—Ni1—C17—O1	−75.39 (18)	C23—C22—C21—C20	0.8 (6)
N3—Ni1—C17—O1	103.90 (18)	N2—C3—C5—C6	−74.9 (4)
O2—Ni1—C17—O1	−170.0 (3)	C4—C3—C5—C6	160.9 (4)
N4—Ni1—C17—C18	81.2 (15)	C9—N3—C6—C5	76.9 (4)
N2—Ni1—C17—C18	−91.2 (14)	Ni1—N3—C6—C5	−47.7 (4)
N1—Ni1—C17—C18	179 (100)	C9—N3—C6—C7	−161.4 (3)
N3—Ni1—C17—C18	−2.0 (14)	Ni1—N3—C6—C7	74.1 (3)
O2—Ni1—C17—C18	84.1 (14)	C9—N3—C6—C8	−43.6 (4)
O1—Ni1—C17—C18	−105.9 (15)	Ni1—N3—C6—C8	−168.2 (3)
O2—C17—C18—C19	−143.1 (3)	C3—C5—C6—N3	64.5 (4)
O1—C17—C18—C19	37.1 (4)	C3—C5—C6—C7	−54.8 (4)
Ni1—C17—C18—C19	138.0 (13)	C3—C5—C6—C8	−173.9 (3)
O2—C17—C18—C23	32.3 (4)		

### Hydrogen-bond geometry (Å, °)

**Table:**

*D*—H···*A*	*D*—H	H···*A*	*D*···*A*	*D*—H···*A*
N1—H1C···O5^i^	0.93	2.29	3.094 (9)	144
N2—H2C···O6'^ii^	0.93	2.03	2.952 (10)	173
O4—H4···O1W	0.84	1.76	2.572 (4)	162
O1W—H2W···O8	0.86 (1)	2.13 (3)	2.827 (5)	138 (4)
O1W—H1W···O1^iii^	0.86 (1)	1.89 (2)	2.734 (3)	166 (4)

Symmetry codes: (i) *x*, −*y*+1/2, *z*+1/2; (ii) −*x*+1, −*y*, −*z*; (iii) *x*, −*y*+1/2, *z*−1/2.
